# The Possible Physical Barrier and Coastal Dispersal Strategy for Japanese Grenadier Anchovy, *Coilia nasus* in the East China Sea and Yellow Sea: Evidence from AFLP Markers

**DOI:** 10.3390/ijms16023283

**Published:** 2015-02-03

**Authors:** Zhi-Qiang Han, Gang Han, Zhi-Yong Wang, Tian-Xiang Gao

**Affiliations:** 1 Fishery College, Zhejiang Ocean University, Zhoushan 316022, China; E-Mail: hanzq@zjou.edu.cn; 2 Institute of Evolution & Marine Biodiversity, Ocean University of China, Qingdao 266003, China; E-Mail: hangang@cafs.ac.cn; 3 Fishery College, Jimei Universtiy, Xiamen 361021, China; E-Mail: zywang@jmu.eu.cn

**Keywords:** *Coilia nasus*, phyiscla barrier, East China Sea, coastal dispersal pattern

## Abstract

In order to ascertain the taxonomic status of the Ariake Sea population of Japanese grenadier anchovy, *Coilia nasus*, and assess the contemporary possible genetic barrier between the west and east coastal waters of the East China Sea, we used amplified fragment length polymorphism (AFLP) markers to detect the genetic structure of *C. nasus*, in the East China Sea and Yellow Sea. Eighty-one individuals of *C. nasus* were collected from five locations and 12 individuals of *Coilia mystus* were sampled from the Yangtze River Estuary. A total of 371 loci were detected by five primer combinations, 310 of which were polymorphic (83.56%). Analysis of molecular variation (AMOVA) and pairwise fixation index (*F*_ST_) revealed significant genetic differentiation among five samples, indicating limited gene flow among populations. The dendrogram for populations by neighbor-joining (NJ) cluster analysis provided evidence of a clear relationship between genetic and geographic patterns, supporting significant genetic differentiation between China coastal populations and Ariake Sea populations. Compared to the genetic divergence between *C. nasus* and *C. mystus*, the level of genetic differentiation between China and the Ariake Sea populations of *C. nasus* is obvious below the species level, indicating isolated populations of *C. nasus* in the Ariake Sea. Isolation by distance analysis revealed that direct ocean distance with deep-water at the continental slope and high salinity between west and east coastal waters of the East China Sea served as major physical barrier to *C. nasus*, supporting the coastal dispersal pattern in this estuarine species, and rejecting offshore dispersal strategy.

## 1. Introduction

A continuing challenge in evolutionary biology of marine species is to understand the process by which populations become genetically distinct, in a fluid ecosystem that tends to homogenize populations [1]. Genetic structure in a marine environment without any obvious physical boundary to gene flow has been explained by a number of mechanisms (reviewed in [2]). Some factors that cause population subdivision in marine organisms may be generally important either singly or in combination [3,4,5,6,7]. Recently comparative phylogeographical studies conducted in the East China Sea focused on the role of Pleistocene isolation in shaping the genetic structure of marine organisms (reviewed in [8]). The geographical genetic patterns of genetic structure within a species were shaped not just by the historical connectivity, but also by the modern connectivity among populations [9,10,11]. However, the role of candidate contemporary genetic barriers to marine species between the west and east coastal waters of the East China Sea was ignored. Except the Yangtze River outflow [10], no other possible contemporary genetic barrier to gene flow is clearly identified in the East China Sea. To understand the evolutionary mechanisms shaping the genetic population structure in the East China Sea, it is necessary to detect contemporary genetic barriers for marine species by molecular markers with fast mutation rates.

Japanese grenadier anchovy, *Coilia nasus*, is a small to moderate-sized fish species belonging to the family Engraulidae, and is widely distributed in the Yangtze River and other major rivers, localized in the coastal waters of China, and Korea as well as the Ariake Sea of southwestern Japan [12,13]. Based on earlier morphological and ecological studies of the grenadier anchovy, two ecotypes of *C. nasus* have been found in the middle and lower reaches of the Yangtze River basin: A resident population and an anadromous population (reviewed in [14]). The former population is composed of a freshwater resident population, *Coilia brachygnathus*, and a landlocked population,* C. nasus* *taihuensis*. Before sexually mature, the anadromous population of *C. nasus* grows in coastal waters near the estuary, and then run several kilometers up the rivers and spawns in the fresh water at the beginning of spring [15]. The eggs float down and hatch near the river mouth, and adults of *C. nasus* live in the marine environment [16]. The distribution of* C. nasus* in marine waters was limited by the water depth (<60 m), according to the spatial structures of fish communities on the continental shelf of the East China Sea. Fish communities in East China were classified into the three groups. *C. nasus* belonged to the offshore community [17]. The genetic status of these populations of *C. nasus* in China coastal waters were well studied by molecular markers and morphological characters [14,18,19,20], indicating limited gene flow among populations in China. However, only a few genetic studies have considered the genetic status of the Ariake Sea population of *C. nasus* in Japan. Yuan and Qin (1985) considered individuals of *C. nasus* from the Ariake Sea as a unique population, which distinguished it from the Chinese population on the basis of morphological differences [21]. Based on inter-simple sequence repeat (ISSR) markers, individuals of *C. nasus* showed reciprocal monophyly in the populations between China and the Ariake Sea of Japan [19], indicating possible cryptic species in the Ariake Sea. However, the results from the mitochondrial DNA (mtDNA) control region revealed two distinct lineages, which were sympatric in Dongying, Zhenjiang and Natong populations [20]. The lack of an outgroup in the ISSR study and conflicting results between ISSR and mtDNA markers make it difficult to determine the genetic status of the Ariake Sea population. The previous studies raised a series of questions: What is the true genetic relationship between China and the Ariake Sea populations of *C. nasus*? Which factor(s) might drive this phylogeographic pattern of *C. nasus*? Among the molecular markers available for analysis of population genetic structure, amplified fragment length polymorphism (AFLP) markers are characterized as suitable identification of individuals and populations due to their hypervariability, abundance and neutrality [22,23]. To answer these questions, AFLP markers were therefore applied to determine the degree of genetic differentiation between geographical groups with* C. mystus* as an outgroup, and to elucidate genetic barriers in this species. The coastal dispersal model proposed by the mtDNA marker was also tested in this study.

## 2. Results and Discussion

### 2.1. Results

A total of 371 loci were detected from 90 individuals of *C. nasus* and* C. mystus* by the five primer combinations, 310 of which (83.56%), were polymorphic (Table 1). The average number of bands scored per primer pair was 74.2, ranging from 42 to 105. The number of polymorphic loci amplified by each primer combination over all populations ranged from 37 to 87, with the average of 62 polymorphic loci per prime combination (Table 2).

**Table 1:** Parameters of genetic diversity for populations of *Coilia nasus* and *Coilia mystus.*

Populations	Number of Individuals	Date of Collection	Number of Loci	Number of Polymorphic Loci	Proportion of Polymorphic Loci	Nei’s Genetic Diversity
*Coilia mystus*	12	May 2005	286	193	67.48%	0.1435
Zhenjiang (ZJ)	20	July 2004	282	143	50.71%	0.1016
Nantong (NT)	17	October 2004	279	125	44.80%	0.0808
Dongying (DY)	20	May 2004	272	116	42.65%	0.0750
Kashima (KA)	12	March 2003	230	69	30.00%	0.0554
Yanagawa (YA)	9	March 2003	231	73	31.60%	0.0584
Total	93	–	371	310	85.36%	–

**Table 2:** Number of bands generated by primer combinations.

Primer Combinations	E-AGA/M-CAG	E-AGA/M-CTA	E-AGG/M-CAC	E-ACC/M-CAG	E-AGT/M-CTT	Total
Number of loci	105	87	72	42	65	371
Number of polymorphic loci	87	75	59	37	52	310
Proportion of polymorphic loci	85.71%	86.20%	81.94%	88.10%	80.00%	83.56%

Population of *C. mystus* showed higher genetic diversity than populations of *C. nasus* (Table 1). The proportion of polymorphic loci and Nei’s genetic diversity for *C. mystus* were 67.48% and 0.1435, respectively. Among five populations of *C. nasus*, the proportion of polymorphic loci and Nei’s genetic diversity declined from the Yangtze River to the Ariake Sea along the coastline. The population of *C. nasus* with the highest proportion of polymorphic loci (50.71%) and Nei’s genetic diversity (0.1016) was population Zhenjiang in the Yangtze River, whereas that with the lowest value was population Kashima in the Ariake Sea. The proportion of polymorphic loci and Nei’s genetic diversity of population Kashima was 30.00% and 0.0554, respectively. The genetic diversity of the Dongying population in the Yellow River showed a median value between the Yangtze River and the Ariake Sea populations.

**Figure 1:**
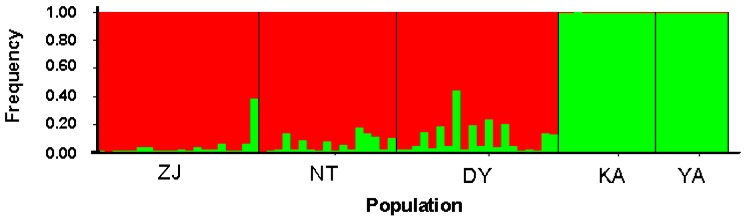
Spatial genetic structure according to a Bayesian assignment probability analysis using the program STRUCTURE 2.3. *K* = 2 appeared to be the optimal number of clusters by showing the Δ*K* at its peak.

**Figure 2:**
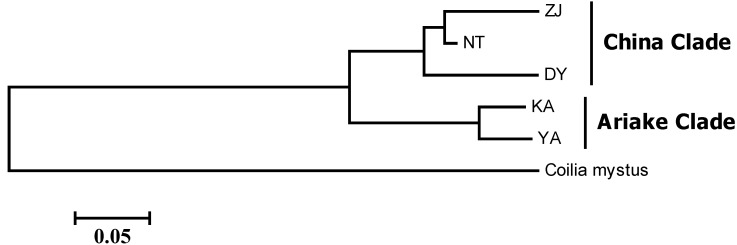
The NJ tree based on the genetic distance.

**Figure 3:**
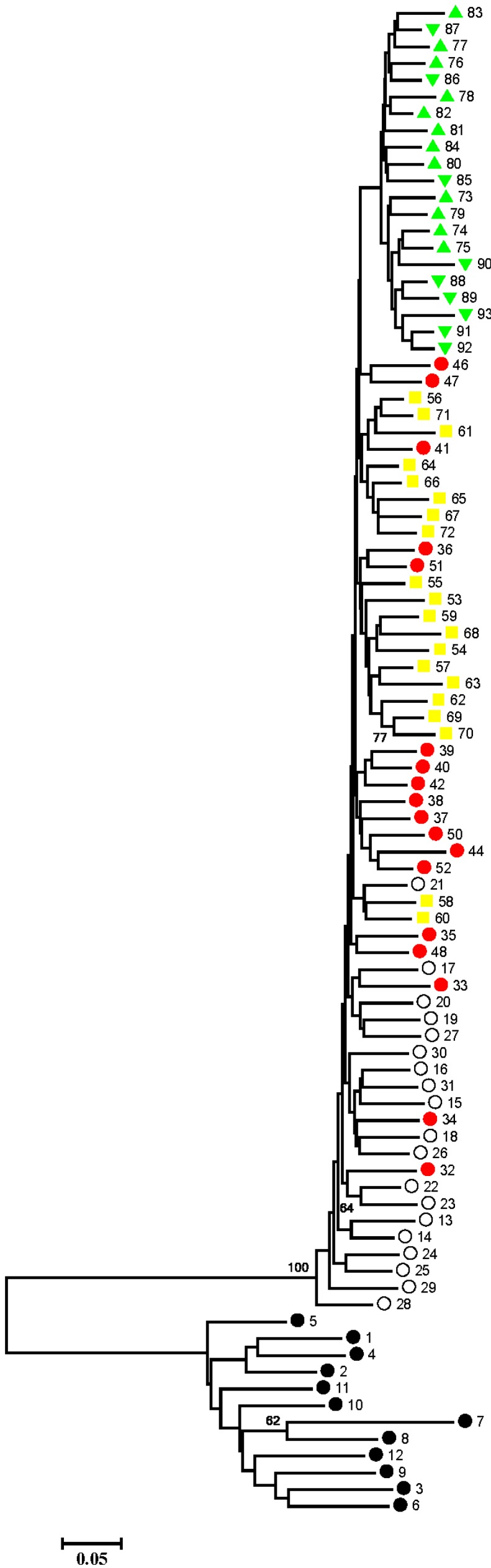
NJ tree of all individuals based on the genetic distance (Black circles, 1–12: *Coilia mystus*; White circles, 13–32: Zhenjiang; Red circles, 33–52: Nantong; Yellow squares, 53–72: Dongying; Regular triangles, 73–84: Kashima; Inverted triangles, 85–93: Yanagawa).

The Bayesian population assignment analysis using the software STRUCTURE revealed the significant separation of the Ariake Sea and Chinese populations. The optimal *K* was 2, which revealed the Ariake Sea cluster and China cluster (Figure 1). According to the AMOVA result, overall genetic differentiation among five populations of *C. nasus* was large and significant (*F*_ST_ = 0.1777, *p* < 0.001) (Table 3), suggesting significant genetic differentiation among localities. The hypothetical grouping of populations by Bayesian assignment analysis were also examined in AMOVA, one group representing the two samples in the Ariake Sea, and another group consisting of three China coastal samples. AMOVA results of the two groups revealed that 14.84% of the total molecular variance can be attributed to regional differences between the two groups, while 9.01% was apportioned among the population within the group and 76.15% among individuals within the population (Table 3). Genetic differentiation between groups is higher than that among populations within groups (fixation index between groups* F*_CT_ = 0.1484, *p* < 0.001). Moreover, pairwise fixation index (*F*_ST_) values among populations of *C. nasus* were also significant (*p* < 0.001), ranging from 0.0732 to 0.2749 (Table 4). These analyses indicated that several distinct populations of *C. nasus* existed in the study area. Additionally, pairwise *F*_ST_ analysis indicated the largest genetic difference among populations existed between Zhenjiang and Yanagawa populations (*F*_ST_ = 0.2749, *p* < 0.01), whereas the genetic difference between Yanagawa and Kashima was the smallest (*F*_ST_ = 0.0732, *p* < 0.01). Genetic distance analysis suggested that the Zhenjiang and Kashima populations were the most different genetically (*D* = 0.1337), whereas the most similar populations were Yanagawa and Kashima (*D* = 0.0551) (Table 4). Further, the dendrogram for populations by NJ cluster analysis provided evidence of a clear relationship between genetic and geographic patterns; in particular, the most divergent samples, Kashima and Yanagawa in the Ariake Sea, were genetically well differentiated from the sample collected from China coastal sites, whereas the samples Zhenjiang and Nantong in the Yangtze River, the geographically nearest, were grouped together (Figure 2). Although most of individuals from the same site clustered first together, the neighbor-joining (NJ) cluster analysis for individuals, based on genetic distances, showed shallow topology and no distinct clade (Figure 3).

**Figure 4:**
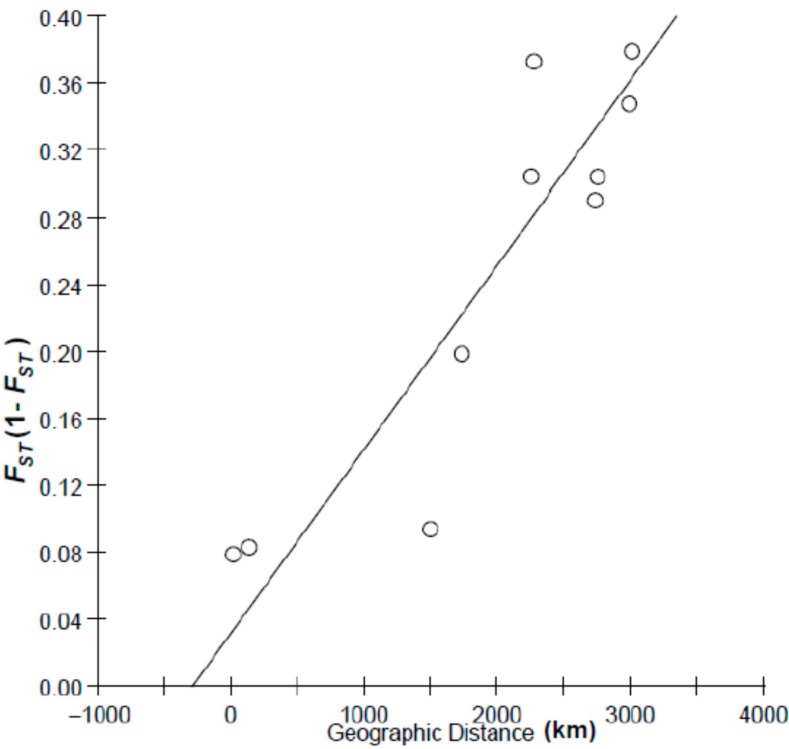
Plot of pairwise estimates of *F*_ST_/(1 − *F*_ST_)* vs.* coastal distance between samples.

**Figure 5:**
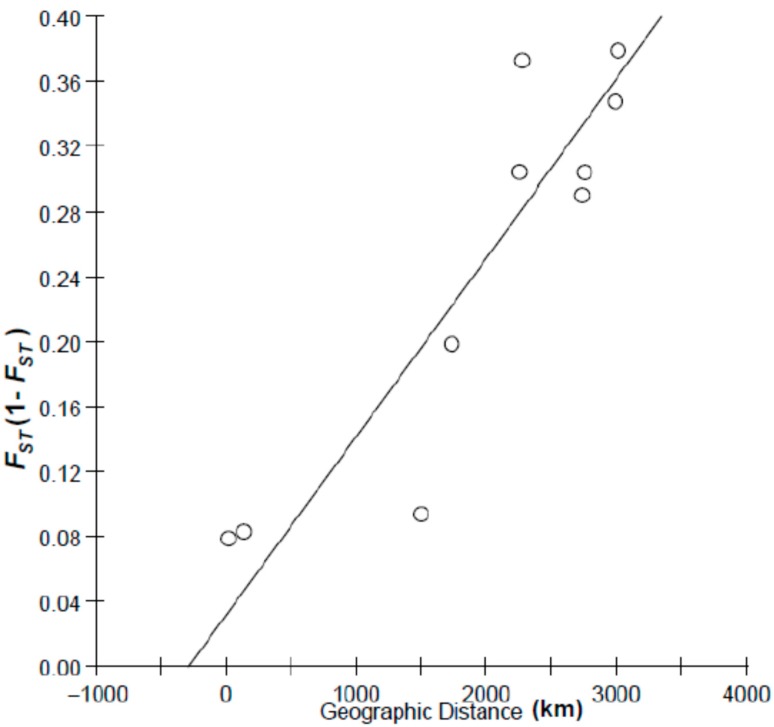
Plot of pairwise estimates of *F*_ST_/(1 − *F*_ST_)* vs.* oceanic distance between samples.

To test the relationship between geographic distance and genetic distance, two patterns of geographic distance (coastal distance and oceanic distance) were used in isolation by distance (IBD) analysis. Among sample sites, the Mantel test indicated a more close relationship (*r* = 0.90) between* F*_ST_/(1 − *F*_ST_) and coastal distance than that between *F_ST_*/(1 − *F_ST_*) and oceanic distance (*r* = 0.50), indicating isolation by costal distance (Figure 4 and Figure 5), with coastal distance explaining 90% of the variation in genetic differentiation for species.

**Table 3:** Analysis of molecular variance (AMOVA) based on the AFLP markers.

Source of Variation	Variance Components	Percentage of Variance	*F*/φ-Statistics	*p*
**One gene pool**				
Among populations	3.6003	17.77	0.1777	0.00
Within populations	16.6565	82.23		
**Two gene pools (DY, NT, ZJ) (Ka, Ya)**				
Between groups	3.2468	14.84	0.1484	0.00
Among populations within groups	1.9707	9.01	0.1058	0.00
Within populations	16.6565	76.15	0.2385	0.00

**Table 4:** Genetic distance *D* (above) and pairwise *F*_ST_ (below) between populations.

Populations	*Coilia mystus*	Zhenjiang	Nantong	Dongying	Kashima	Yanagawa
*Coilia mystus*	–	0.6406	0.6360	0.6404	0.6616	0.6550
Zhenjiang	0.6715	–	0.1102	0.1148	0.1337	0.1246
Nantong	0.6880	0.0746	–	0.0920	0.1141	0.1002
Dongying	0.6946	0.1660	0.0789	–	0.0962	0.0841
Kashima	0.7136	0.2580	0.2203	0.2333	–	0.0551
Yanagawa	0.7093	0.2749	0.2302	0.2717	0.0732 *	–

* Significant *p* < 0.05.

### 2.2. Discussion

Previous genetic studies of Japanese grenadier anchovy* C. nasus* based on the ISSR and mtDNA markers revealed different genetic scenarios [19,20]. ISSR markers of *C. nasus* showed reciprocal monophyly in populations between China and the Ariake Sea [19]. However, two distinct lineages were detected by the mtDNA control region sequence, which coexisted in the Dongying sample [20]. The conflicting results between previous ISSR and mtDNA markers raised the question whether the presence of divergent mitochondrial lineages in the same sample was a result of secondary contact after an extended period of isolation and/or the presence of two cryptic species. The genetic status of the Ariake Sea population was well assessed by AFLP markers in the present study. Compared to the genetic distance between *C. nasus* and *C. mystus*, the level of genetic differentiation between China and the Ariake Sea populations of *C. nasus* is obvious below the species level, indicating new isolated populations. AFLP markers showed similar result with the mtDNA control region [20]. No complete genetic break was revealed between the China and the Ariake Bay populations based on the Bayesian population assignment analysis, which agreed with the mtDNA control region result [20]. However, the topology of individuals NJ tree partly agreed with the previous results based on ISSR markers [19], which showed reciprocal monophyly in populations between China and the Ariake Bay. In the present study, the topology of individuals NJ tree were shallow and no distinct clade was detected from the NJ tree. According to the present AFLP result, the presence of divergent mitochondrial lineages in the Dongying, Zhenjiang and Nantong samples was a result of secondary contact.

Genetic population study commonly assumes that significant differentiation at neutral markers correspond to restricted dispersal between geographic localities [24]. To explain the genetic phylogeography of *C. nasus*, previous mtDNA control region study revealed that sea-level fluctuations during the late Pleistocene caused the isolation of the Ariake Sea from other populations, resulting in the two distinct mitochondrial lineages [20]. The marginal seas of the Western Pacific separating Asia from the Pacific were thought to be affected greatly by Quaternary glaciations. In each of the Quaternary glaciation events with decline in sea levels of 120–140 m, the gene flow between marine organisms in the marginal seas and Pacific Ocean was interrupted during this lowering of the sea level at glacial maxima and re-established during the flooding of the interglacial periods. The lower sea level acted as historical barrier for marine organisms, potentially aiding allopatric diversification or genetic differentiation of populations (reviewed in [8]).

Besides historical factors, contemporary environmental factors also play a pivotal role in shaping the present-day phylogeographic pattern. Considering the population cluster tree and Bayesian analysis, the Dongying population may be genetically intermediate between Nantong/Zhenjiang and Ariake Sea samples, which coincide with the previous mtDNA result [20]. The coastal dispersal pattern of *C. nasus* and the ocean distance between the Yangtze River and the Ariake Sea as genetic barrier proposed by previous mtDNA markers were supported by our AFLP results. The plot of *F*_ST_/(1 − *F*_ST_) and geographic distances (coastline distance and ocean distance) revealed a strong pattern of isolation by distance under the coastal dispersal model in *C. nasus*, which indicates that ocean distance separating western and eastern East China Sea is sufficiently large to restrict gene flow between samples. What about the mechanism that the ocean distance performs as genetic barrier to the dispersal of *C. nasus*? Considering the life history of this species and physical environment in the East China Sea, it is reasonable that large ocean distance is a physical barrier for an anadromous and estuarine species. As an estuarine species, *C. nasus* is sensitive to the high salinity (>30) [15]. Additionally, the distribution of* C. nasus* in marine waters was limited by depth (<60 m) [17]. The average depth of East China Sea is about 370 m, with a maximum of 2719 m at the continental slope [10]. Therefore, the high salinity of ocean waters (32–35) and deep depth water (depth > 1000 m) in the direct dispersal route between the Yangtze River estuary and Ariake Sea might form an unsuitable habitat for this species, and prevent offshore dispersal. To choose a suitable habitat, coastal dispersal might be good choice for *C. nasus*.

From our AFLP data and previous ISSR and mtDNA results [19,20], a clear scenario to explain the phylogeographic pattern of this species was inferred. The historical factor of Pleistocene low sea levels caused the genetic isolation between western and eastern East China Sea sites (China coastal waters and Ariake Sea). The previous control region data suggested a divergence time of 107,500 years between the two lineages in the Late Pleistocene [20]. The dating of divergence was consistent with the beginning of Wurm glaciation that might have created a vicariant barrier between populations of Japan and the East China Sea [25]. Since the end of this glacial period when favorable conditions emerged, the Yellow Sea/Yangtze River Estuary were a post-glacial contact zone for two isolated groups and were recolonized by the China isolated group from a western route of dispersion along the China coastline, and Ariake Sea group from an eastern route of dispersion along the Korean Peninsula coastline. The mixture of two groups in the three populations of China and lack of a China group in the Ariake Sea indicated a unidirectional dispersal route from the Ariake Sea along the Korean Peninsula coastline. This south to north unidirectional dispersal route might have caused the northward ocean current, Yellow Sea Warm Current, which facilitates the dispersal of *C. nasus* from the Ariake Sea along the Korean Peninsula coastline. The present study revealed large ocean distance between the Yangtze River Estuary and the Ariake Sea was an effective contemporary physical barrier for estuarine species on both sides of the coastal waters of the East China Sea. This finding answered the unresolved question proposed by previous phylogeographic studies in the Northwestern Pacific. What is the contemporary environmental factor to maintain the genetic differentiation? The large ocean distance is the contemporary environmental factor to maintain the genetic differentiation of some species, especially the estuarine species. We surveyed the public genetic studies of estuarine species in the study area, which supported this inference. For example, three mtDNA distinct lineages were observed in redlip mullet *Chelon haematocheilus*, which was the typical estuarine species in the Northwestern Pacific [26]. The genetic pattern of *C. nasus*, samples of Dandong from northern Yellow Sea near Korean Peninsula coastline and Tianjin from Bohai Sea were likely genetically intermediate between the East China Sea and the Japan coastal populations of *C. haematocheilus*. The three lineages of* C. haematocheilus* were thought to diverge in the three marginal seas during the Pleistocene low sea levels. The large ocean distance was the plausible reason to prevent the dispersal between China coastal waters and Japan coastal waters. Similar results were also observed in *Trachidermus fasciatus* [27]. Two distinct clades (China and Ariake Sea clades) were observed in *T. fasciatus* based on SSR markers and they coexisted in the sample of Rongcheng from the northern Yellow Sea. Historical isolation associated with the present large ocean distance barrier has been the general explanation to elucidate the evolutionary mechanisms shaping the genetic population structure for these species.

This general explanation can also be used to explain the species divergence between *Lateolabrax japonicus* and *L. maculates* [28], which were commonly found in Japan and China estuarine and coastal waters, respectively. The historical events created a vicariant barrier between China and Japan costal waters, isolating the origin populations of *Lateolabrax* species. The ocean distance between China and Japan served as a physical barrier to prevent secondary contact for *Lateolabrax* populations after isolation events and create allopatric speciation. Besides estuarine species, some phylogeographical pattern of some marine species in the study area can also be interpreted by this general explanation. Two geographic clades (the China and Japan clades) were detected among populations of white croaker *Pennahia argentata* by AFLP markers and mtDNA control region [29,30]. Historical isolation during Pleistocene low sea level and the large ocean distance preventing secondary contact after the isolation event were also the reasonable interpretation to explain the presence of two allopatric clades in white croaker. Population genetic studies of Patagonian toothfish populations in the Southwest Atlantic Ocean also showed results similar to our study [31]. The Patagonian toothfish is distributed across most islands, sea mounts and shelf areas in the sub-Antarctic waters of the Atlantic, Indian and Pacific Ocean sectors of the Southern Ocean. MtDNA data revealed deep-water troughs and distance between sites contributing to genetic differentiation in this species.

However, the ocean distance serving as physical barrier is only applied in estuarine species, not in pelagic species. The genetic study of typical pelagic species Japanese anchovy *Engraulis japonicus* and Japanese Spanish mackerel *Scomberomorus niphonius* in the Northwestern Pacific revealed no significant genetic differentiation [32,33], indicating invalidity of ocean distance serving as physical barrier. Therefore, our finding implied that evaluation of the influence of environmental factors on genetic structure should consider the character of the species.

## 3. Materials & Methods

### 3.1. Sample Collection

**Figure 6:**
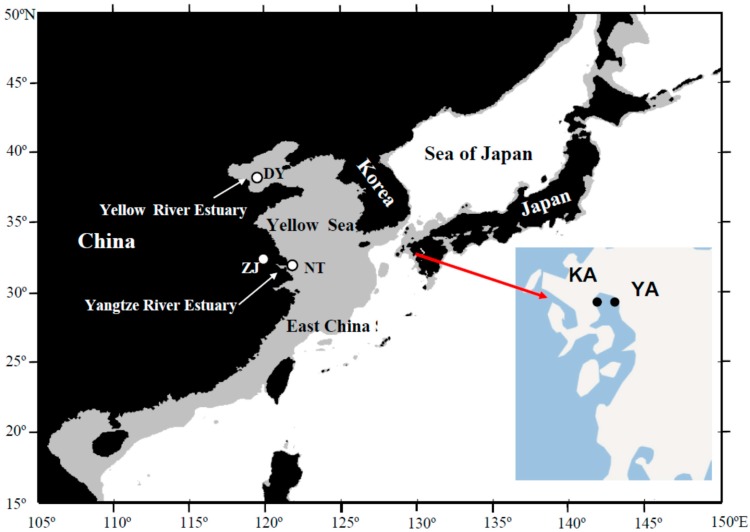
Map of the *Coilia nasus* collection sites.

A total of 81 individuals of *C. nasus* were collected in five sites along Chinese and Japanese coastal waters, including two locations (Zhenjiang and Nantong) from the Yangtze River, one location (Dongying) in the Yellow River Estuary, and two locations (Kashima and Yanagawa) from the Ariake Sea (Table 1; Figure 6). In addition, twelve individuals of *C. mystus* were collected from the Yangtze River Estuary. Muscle samples were obtained and preserved in 95% ethanol or frozen for DNA extraction after specimen identification.

### 3.2. AFLP Analysis

Genomic DNA was isolated from muscle tissue by proteinase K digestion followed by a standard phenol–chloroform method. DNA was subsequently resuspended in 100 μL of TE buffer (10 mmol/L Tris–HCl, 1 mmol/L EDTA, pH = 8.0). Procedures of AFLP were essentially based on Vos* et al.* (1995) and Wang* et al.* (2000) [22,34]. PCR products were run on 6.0% denaturing polyacrylamide gel electrophoresis (PAGE) for 2.5 h at 50 °C on the Sequi-Gen GT Sequencing Cell (Bio-Rad, Hercules, CA, USA), and finally detected using the silver staining technique modified from Merril* et al.* (1979) [35]. Sequences of AFLP adapters and primers are listed in Table 5. Five primer combinations (E-AGA/M-CAG, E-AGA/M-CTA, E-AGG/M-CAC, E-ACC/M-CAG, E-AGT/M-CTT) were chosen for AFLP analysis (Table 5).

**Table 5:** Adaptor and primer sequences used in AFLP analysis.

Primer	Sequence
**Adapters**	
*Eco*RI-adapter	5'-CTCGTAGACTGCGTACC-3'
	5'-AATTGGTACGCAGTCTAC-3'
*Mse*I-adapter	5'-GACGTGAGTCCTGAG-3'
	5'-TACTCAGGACTCAT-3'
**Pre-Amplification Primer**	
*Eco*RI	5'-GACTGCGTACCAATTC-3'
*Mse*I	5'-GATGAGTCCTGAGTAA-3'
**Selective Amplification Primer**	
E-AGA/M-CAG	5'-GACTGCGTACCAATTCAGA-3'
	5'-GATGAGTCCTGAGTAACAG-3'
E-AGA/M-CTA	5'-GACTGCGTACCAATTCAGA-3'
	5'-GATGAGTCCTGAGTAACTA-3'
E-AGG/M-CAC	5'-GACTGCGTACCAATTCAGG-3'
	5'-GATGAGTCCTGAGTAACAC-3'
E-ACC/M-CAG	5'-GACTGCGTACCAATTCACC-3'
	5'-GATGAGTCCTGAGTAACAG-3'
E-AGT/M-CTT	5'-GACTGCGTACCAATTCAGT-3'
	5'-GATGAGTCCTGAGTAACTT-3'

### 3.3. Data Analysis

AFLP bands were scored for presence (1) or absent (0), and transformed into 0/1 binary character matrix. Clearly readable AFLP bands were scored. The scoring process was performed visually three times to help ensure correct genotyping. Negative controls were run at each step of the AFLP process to check for exogenous contaminations. Samples with poor DNA quality,* i.e.*, with fragmented genomic DNA on agarose gel, were not used. Individuals with odd profiles,* i.e.*, where most of the bands are not observed in other individuals, were discarded. Proportion of polymorphic loci, Nei’s genetic diversity were calculated by POPGEN (http://cc.oulu.fi/~jaspi/popgen/popgen.htm). Similarity indices were calculated using the formula *S* = 2*N*_ab_/(*N*_a_ + *N*_b_), genetic distances between individuals were computed using the formula *D* = −ln*S* [36]. Genetic relationships among populations were estimated by constructing NJ tree based on genetic distance *D* in Mega 5.0. Population structure of *C. nasus* was investigated using the molecular variance software package (AMOVA) and *F*-statistics in AFLP-SURV [37]. The spatial genetic structure was examined using the Bayesian assignment probability test in the program STRUCTURE 2.3 [38]. This program uses a Bayesian approach to generate posterior probabilities of assignment of individuals to each of a given number of populations. The procedure of AFLP markers analysis in the program STRUCTURE was followed Falush* et al.* [39].

To test for isolation by distance [40], pairwise values of *F*_ST_/(1 − *F*_ST_) were plotted against geographical distance (one-dimensional stepping-stone model) between sample sites of *C. nasus*. The strength and significance of the relationship between genetic distances and geographic distances was assessed using reduced major axis regression and Mantel tests using IBDWS (Isolation by distance web service at http://ibdws.sdsu.edu/~ibdws/). Geographical distances among samples were measured both following the coastline (coastal distances = CD) and by shortest distance across open waters (ocean distances = OD). Under a coastal model of dispersion, distances between Nantong/Zhenjiang and Ariake Bay samples are longer than under the oceanic model.

## 4. Conclusions

Our results assessed the level of genetic differentiation between China and Ariake Sea populations. We confirmed the coastal dispersal model in *C. nasus* and the contemporary physical barrier for estuarine species proposed by mtDNA results. The south to north unidirectional dispersal route of *C. nasus* from the Ariake Sea to Yellow Sea was our new finding. The coastal dispersal model might be one common dispersal pattern for estuarine species. The present study sheds light on some of the evolutionary mechanisms underlying the genetic differentiation for estuarine species in the Northwestern Pacific.
